# Fluid dynamic analysis in predicting the recanalization of intracranial aneurysms after coil embolization – A study of spatiotemporal characteristics

**DOI:** 10.1016/j.heliyon.2023.e22801

**Published:** 2023-12-20

**Authors:** Jing Liao, Kouichi Misaki, Tekehiro Uno, Iku Nambu, Tomoya Kamide, Zhuoqing Chen, Mitsutoshi Nakada, Jiro Sakamoto

**Affiliations:** a Division of Transdisciplinary Sciences, Graduate School of Frontier Science Initiative, Kanazawa University, Ishikawa, Japan; b Department of Neurosurgery, Kanazawa University, Ishikawa, Japan; c Department of Nuclear Medicine, Kanazawa University, Ishikawa, Japan; d Division of Mechanical Science and Engineering, Graduate School of Natural Science and Technology, Kanazawa University, Kanazawa, Ishikawa, Japan

**Keywords:** CFD, flow pattern, Intracranial aneurysm, Pressure difference

## Abstract

**Purpose:**

Hemodynamics play a key role in the management of cerebral aneurysm recanalization after coil embolization; however, the most reliable hemodynamic parameter remains unknown. Previous studies have explored the use of both spatiotemporally averaged and maximal definitions for hemodynamic parameters, based on computational fluid dynamics (CFD) analysis, to build predictive models for aneurysmal recanalization. In this study, we aimed to assess the influence of different spatiotemporal characteristics of hemodynamic parameters on predictive performance.

**Methods:**

Hemodynamics were simulated using CFD for 66 cerebral aneurysms from 65 patients. We evaluated 14 types of spatiotemporal definitions for two hemodynamic parameters in the pre-coiling model and five in virtual post-coiling model (VM) created by cutting the aneurysm from the pre-coiling model. A total of 91 spatiotemporal hemodynamic features were derived and utilized to develop univariate predictor (UP) and multivariate logistic regression (LR) models. The model's performance was assessed using two metrics: the area under the receiver operating characteristic curve (AUROC) and the area under the precision-recall curve (AUPRC).

**Results:**

Different spatiotemporal hemodynamic features exhibited a wide range of AUROC values ranging from 0.224 to 0.747, with 22 feature pairs showing a significant difference in AUROC value (P-value <0.05), despite being derived from the same hemodynamic parameter. PD_ave,q1_ was identified as the strongest UP with AUROC/AUPRC values of 0.747/0.385, yielding sensitivity and specificity value of 0.889 and 0.614 at the optimal cut-off value, respectively. The LR model further improved the prediction performance, having AUROC/AUPRC values of 0.890/0.903. At the optimal cut-off value, the LR model achieved a specificity of 0.877, sensitivity of 0.719, outperforming the UP model.

**Conclusion:**

Our research indicated that the characteristics of hemodynamic parameters in terms of space and time had a significant impact on the development of predictive model. Our findings suggest that LR model based on spatiotemporal hemodynamic features could be clinically useful in predicting recanalization after coil embolization in patients, without the need for invasive procedures.

## 1 Introduction

The coil embolization imposes a comparatively reduced physical burden on the patient when compared with surgical clipping; however, the notable concerns encompass recanalization and the necessity for subsequent aneurysm retreatment [1,2]. Previous studies have observed recanalization in approximately 10 %–25 % of coil embolic aneurysms during follow-up [3].

Based on hemodynamic parameters from computational fluid dynamics (CFD), quantified models have been developed to predict aneurysmal growth, rupture, and recanalization [4,5]. In previous studies, both spatiotemporally averaged and maximal definitions for the hemodynamic parameters were found to build predictive models for aneurysm recanalization. One study developed a predictive parameter by combining maximal and average hemodynamic, morphological, and clinical scalars [6]. Another study revealed significant differences in spatiotemporally averaged aneurysmal residual flow volume and maximal force between recanalized and stable aneurysms after coil embolization [7]. Recent studies have conducted a comprehensive analysis of the risk factors for aneurysmal recanalization using a virtual post-coiling model (VM) created by cutting the aneurysm from the pre-coiling model [1,2,8,9]. In comparison with other researched morphological and hemodynamic parameters, pressure difference (PD) in VM was the strongest predictor of aneurysmal recanalization. However, the spatiotemporal characteristics of hemodynamic parameters have not been considered by these models.

The present study aimed to examine the influence of spatiotemporal hemodynamic characteristics on predictive accuracy. We identified 14 spatiotemporal features from each hemodynamic parameter, resulting in a total of 91 spatiotemporal hemodynamic features. We assessed the predictive performance of the identified features for aneurysm recanalization using univariate and multivariate analyses.

## 2 Method

### 2.1 Patients

Definitions of recanalized/stable aneurysms and the inclusion criteria of patients could be found in our previous works [1,2,8,9]. The final analysis included a cohort of 66 intracranial aneurysms derived from 65 patients who underwent endovascular treatment for aneurysms within the timeframe of January 2007 to December 2020, comprising nine recanalized aneurysms and 57 stable aneurysms. Post-treatment, the enrolled patients underwent systematic biannual follow-ups utilizing magnetic resonance angiography. In instances where magnetic resonance imaging raised suspicion of recanalization, confirmation was sought through digital subtraction angiography. Endovascular intervention was administered as dictated by clinical necessity.

### 2.2 Fluid dynamic analysis

The methodology for conducting CFD based on medical imaging can be found in our previously published studies [1,2,8,9]. Two complete cardiac cycles, equivalent to a duration of 1.8s, were computed and employed as the basis for this study.

### 2.3 Features deviation

Morphological parameters were assessed utilizing 3D-RA data, encompassing measurements of the maximum size, neck width, height, as well as the area of the posterior communicating (Pcom), aneurysm neck and inlet [1,10]. Aspect ratio was defined as the quotient of the maximum perpendicular height to the neck diameter [11,12]. Bottleneck factor was defined as the ratio of the dome width to the neck diameter [13]. Size ratio was defined as the ratio of the maximum aneurysm height to the parent vessel diameter [14]. Area ratio was articulated as the proportion of the aneurysm neck area to the area of the aneurysm inlet within the parent artery [15]. VER was represented as the proportion of the aneurysm volume to the volume occupied by the coil [16].

Dimensionless hemodynamic parameters were employed in the analysis, enabling the construction of a model that was not contingent on patient-specific inflow rates [1,2,6,8]. Hemodynamic parameters in pre-coiling model included velocity in aneurysm dome (volvel), wall shear stress (WSS), static pressure (P) and dynamic pressure (Pdyn) at aneurysm neck plane, which were normalized using surface-averaged velocity, WSS and P at aneurysm inlet in parent artery. Inflow rate ratio (FR) was defined as the ratio of the inflow rate at the neck plane to the flow rate at the inlet plane [1]. In VM, PP was defined as pressure change between aneurysm neck and inlet, whereas pressure difference (PD) was delineated as the quotient of PP to Pdyn at the inlet of the aneurysm, as described by following equation (Eqn. (1)):

(Eqn. 1) $$PD=\frac{P-P_{inlet}}{\frac{1}{2}\rho v_{inlet}^{2}}$$

where v_inlet_ and P_inlet_ indicated spatiotemporally averaged velocity and pressure at the aneurysm inlet plane; ρ is the blood density, 1100 kg/m^3^ [1,2,6,8].

**Fig. 1:**
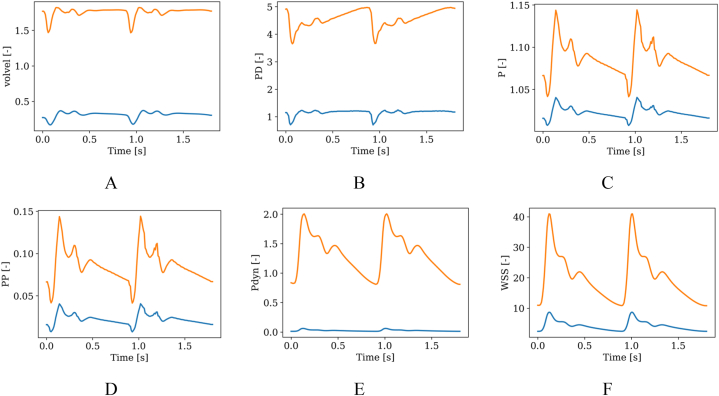
Temporal profiles of spatially averaged (blue) and maximal (orange) values for six parameters: A. volvel, B. PD, C. P, D. PP, E. Pdyn and F. WSS. (For interpretation of the references to colour in this figure legend, the reader is referred to the Web version of this article.)

**Fig. 2:**
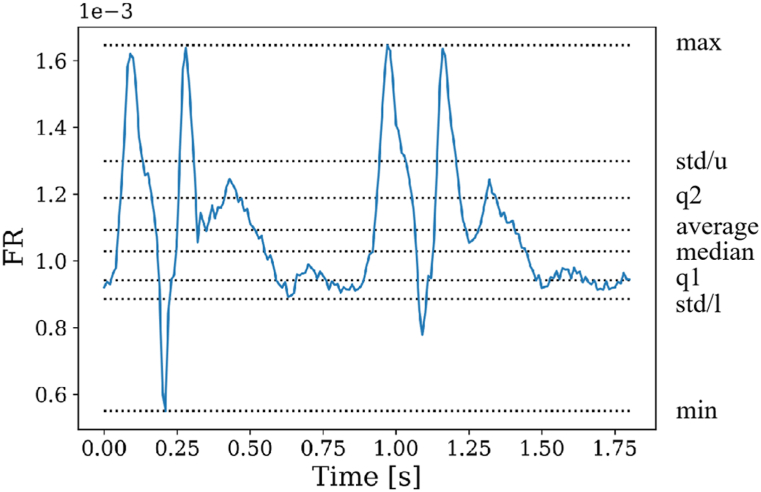
Temporal features derived from time series data of FR: maximum (max), minimum (min), average, median, 25th percentiles (q1), 75th percentiles (q2), upper standard deviation (std/u), lower standard deviation (std/l).

In this study, we established 14 spatiotemporal features for each hemodynamic parameter as shown in Table 1. Spatial features were defined using spatially averaged or maximal hemodynamic values, denoted as X_ave_ and X_max_, where X represented volvel, PD, P, PP, Pdyn, and WSS. On the other hand, FR was solely defined as a mass flow rate variable over time. Fig. 1 A-F depict the temporal profiles of X_ave_ and X_max_ for one patient, revealing that both profiles exhibited a similar trend, but with quantitative differences. To capture the temporal characteristics of the data, we derived seven features (a, median, min, max, std, q1, and q2), as illustrated in Fig. 2.

**Table 1:** 14 spatiotemporal features of hemodynamic parameter X.

Spatially	Temporally						
	average	median	q1	q2	maximum	minimum	std
average	X_ave,a_	X_ave, median_	X_ave,q1_	X_ave,q2_	X_ave,max_	X_ave,min_	X_ave,std_
maximum	X_max,a_	X_max, median_	X_max,q1_	X_max,q2_	X_max,max_	X_max,min_	X_max,std_

### 2.4 Statistical analysis

All continuous parameter values were presented in the format of mean ± standard deviation. The Levene test was employed to evaluate the equality of variances between the two groups with continuous variables [76,77]. Differences in variables between the two groups were examined using Welch's *t*-test in the case of data with unequal variance, or *t*-test for data with equal variance. For categorical variables, the assessment of significant differences between groups was conducted using a chi-square test. A significance level of P-value <0.05 was applied to establish statistical significance. The statistical analyses were conducted utilizing Scipy (version 1.9.3) [17].

### 2.5 Predictive models and evaluation

To assess the predictive capacity of the 91 derived spatiotemporal hemodynamic features, we conducted both univariate predictor (UP) and multivariate logistic regression (LR) analyses. In the UP analysis, we employed receiver operating characteristic curve (ROC) analysis to ascertain the optimal cut-off value for each feature. In the LR analysis, we initially conducted univariate LR analyses for each spatiotemporal feature. Variables demonstrating a level of significance with P-value <0.05 were retained. Furthermore, we computed Variance Inflation Factor (VIF) values to assess multicollinearity among the selected variables from the univariate LR analysis [18]. Variables with a VIF exceeding 10 were deemed indicative of multicollinearity and excluded from the analysis [6,19]. The subsequent multivariate LR analysis utilized the selected variables from the multicollinearity assessment. We implemented a stepwise selection approach guided by P-values, wherein variables for the multivariate LR analysis were iteratively assessed until achieving a P-value of less than 0.05. Additionally, we conducted the Hosmer-Lemeshow goodness-of-fit test for the final model.

Logistic classification algorithms face challenges in learning when certain classes significantly outnumber others [20]. To address this imbalance, we applied the synthetic minority oversampling technique (SMOTE) to harmonize the class distribution within the dataset [21]. Prior to model training, all parameters were subjected to normalization to achieve a standard deviation of 1 and a mean value of zero.

To account for the imbalanced nature of the dataset, we incorporated the area under the precision-recall curve (AUPRC) alongside the area under the receiver operating characteristic curve (AUROC) as a comprehensive metric for performance assessment [22]. The predictive models were both trained and assessed using the open-source Scikit-learn library (version 1.0.2) [23].

## 3 Results

### 3.1 Statistical analysis for patient population

The patient characteristics for each group are detailed in Table 2. No statistically significant differences were observed in age, aneurysm locations, gender distribution or rupture status between the two groups.

**Table 2:** Comparative analysis of clinical characteristics between patients with stable and recanalized aneurysms.

	Stable (N = 57)	Recanalized (N = 9)	P-value
Age	65 ± 13	64 ± 12	0.984
Sex | Female	51	7	0.653
Rupture status	12	4	0.270
Locations | ICPC	43	8	0.800
Locations | IC paraclinoid	10	1	
Locations | IC-Oph	2	0	
Locations |C1	2	0	

### 3.2 Statistical analysis for hemodynamic and morphological data

A parametric test was conducted on both morphological and hemodynamic features to identify the variables that exhibit statistical significance, as presented in Supplementary Table 1. Table 3 presents the mean and standard deviation values of the morphological and hemodynamic features that displayed statistical significance between recanalized and stable groups. The recanalized group showed higher mean values for all significant morphological parameters in comparison to the stable group.

**Table 3:** Significant morphological and hemodynamic features from parametric test.

	Stable (N = 57)	Recanalized (N = 9)	P-value
*Morphological features*			
Max size [mm]	7.987 ± 3.135	11.344 ± 4.330	0.007
Height [mm]	5.967 ± 2.630	8.444 ± 3.855	0.019
Area Neck [mm^2^]	21.813 ± 13.745	34.336 ± 19.490	0.022
*Hemodynamic features (pre-coiling)*			
volvel_ave,min_	0.327 ± 0.134	0.195 ± 0.107	0.007
volvel_ave,max_	0.513 ± 0.170	0.357 ± 0.193	0.016
volvel_ave,q2_	0.462 ± 0.161	0.316 ± 0.169	0.016
volvel_ave,a_	0.441 ± 0.159	0.299 ± 0.161	0.017
volvel_ave, median_	0.443 ± 0.160	0.303 ± 0.162	0.020
volvel_ave,q1_	0.424 ± 0.158	0.288 ± 0.156	0.021
FR_q1_	0.0007 ± 0.00071	0.00175 ± 0.00349	0.050
*Hemodynamic features (VM)*			
PD_ave,max_	0.488 ± 0.591	1.056 ± 0.779	0.015
PD_ave,std_	0.073 ± 0.031	0.102 ± 0.044	0.017
PD_ave,q2_	0.404 ± 0.615	0.948 ± 0.770	0.022
PD_ave, median_	0.368 ± 0.628	0.922 ± 0.774	0.023
PD_ave,a_	0.361 ± 0.623	0.903 ± 0.765	0.024
PD_ave,q1_	0.330 ± 0.638	0.882 ± 0.774	0.025

The spatiotemporal hemodynamic features derived from identical parameters displayed variations in mean values and standard deviations. Spatially averaged features derived from FR, volvel, and PD demonstrated significant differences between the two groups, whereas P-, PP-, Pdyn-, and WSS-related features did not show significant differences. Furthermore, the recanalized group demonstrated higher mean values for FR and PD-related features, whereas all volvel-related features exhibited lower mean values in recanalized aneurysms compared to the stable group.

### 3.3 Predictive model

a) UP analysis

Performance of UP for aneurysm recanalization using all 91 spatiotemporal hemodynamic features is shown in Supplementary Table 2. Among these parameters, PD-related features exhibited the highest performance and occupied the top nine positions in terms of AUROC value. Notably, spatially averaged features outperformed spatially maximal features, with eight out of top ten features being spatially averaged. PD_ave,q1_ demonstrated the highest AUROC value of 0.747, with sensitivity and specificity values of 0.889 and 0.614, respectively, at the optimal cut-off point. Other top-performing features included PD_ave,a_, PD_ave, median_, PD_ave,q2_, PD_ave,max_, PD_max,std_, PD_ave,min_, PD_ave,std_, PD_max,max_ and P_ave,min_, with AUROC values ranging from 0.743 to 0.641. The lowest AUROC was observed for volvel_ave,min_, with a value of 0.224.

Table 4 presents the results of pairwise comparisons conducted between the 14 spatiotemporal features for each hemodynamic parameter. We found that 22 pairs exhibited a significant difference in AUROC value with a P-value <0.05, despite being extracted from one identical hemodynamic parameter. The predictors that showed such significant difference were derived from volvel, P, and PP.

b) LR analysis

**Table 4:** Spatiotemporal features with significantly different AUROCs as UP.

Feature 1	AUROC 1	Feature 2	AUROC 2	P-value
volvel_max,std_	0.624	volvel_ave,a_	0.285	0.013
volvel_max,std_	0.624	volvel_ave,min_	0.224	0.007
volvel_max,std_	0.624	volvel_ave,q1_	0.288	0.008
volvel_max,std_	0.624	volvel_ave, median_	0.288	0.014
volvel_max,std_	0.624	volvel_ave,q2_	0.287	0.008
volvel_max,std_	0.624	volvel_ave,max_	0.300	0.019
P_ave,a_	0.618	P_max,min_	0.427	0.017
P_ave,min_	0.641	P_max,min_	0.427	0.045
P_ave,q1_	0.639	P_max,min_	0.427	0.008
P_ave, median_	0.624	P_max,min_	0.427	0.010
P_ave,q2_	0.610	P_max,min_	0.427	0.034
P_ave,max_	0.591	P_max,min_	0.427	0.034
PP_ave,a_	0.622	PP_max,std_	0.409	0.000
PP_ave,a_	0.622	PP_max,min_	0.427	0.021
PP_ave,min_	0.618	PP_max,std_	0.409	0.029
PP_ave,q1_	0.641	PP_max,std_	0.409	0.000
PP_ave,q1_	0.641	PP_max,min_	0.427	0.010
PP_ave, median_	0.628	PP_max,std_	0.409	0.001
PP_ave, median_	0.628	PP_max,min_	0.427	0.019
PP_ave,q2_	0.616	PP_max,std_	0.409	0.004
PP_ave,q2_	0.616	PP_max,min_	0.427	0.035

First, we conducted a univariate LR analysis on all 91 features, as shown in Supplementary Table 3, revealing 42 features with P-values less than 0.05. To select features with low multicollinearity, we performed a VIF analysis on these 42 parameters, resulting in a selection of eight parameters with VIF <10 as listed in Supplementary Table 4. Subsequently, we performed multivariate LR analysis and stepwise selection using these eight parameters, ultimately selecting Area Ratio, volvel_ave,q1_, PD_ave,min_, and PD_ave,std_ as the most effective factors for predicting recanalization, as presented in Table 5. PD_ave,std_ exhibited the lowest P-value of 8.172E-07. The Hosmer-Lemeshow goodness-of-fit test yielded a significant result (P-value of 0.909) for the final model, indicating that the model demonstrated a valid and accurate prediction of recanalization outcomes.

c) Performance evaluation

**Table 5:** Features selected by LR analysis.

	5 % CI	95 % CI	Odds Ratio	P-value
Area Ratio	7.207E-07	0.024	0.000	0.001
volvel_ave,q1_	0.001	0.620	0.023	0.025
PD_ave,std_	8.601E+12	9.977E+29	2.929E+21	8.172E-07
PD_ave,min_	3.034	31.963	9.847	1.405E-04

**Fig. 3:**
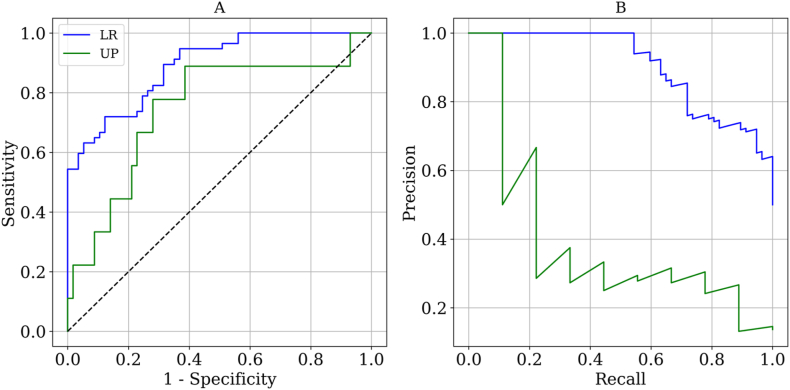
A. ROC and B. PRC analysis of the best UP (PD_ave,q1_) and LR model.

We conducted ROC and PRC analysis on the optimal UP (PD_ave,q1_) and LR model, as shown in Fig. 3 A and B, respectively. The results are detailed in Table 6. Although PD_ave,q1_ was the most effective UP with an AUROC value of 0.747, it was inferior to the result obtained from LR model, which achieved a higher AUROC value of 0.890 with specificity and sensitivity values of 0.877 and 0.719, respectively. Furthermore, a statistically significant discrepancy in AUROC values was noted between the two models, with a P-value below the threshold of 0.05. Additionally, the LR model exhibited a significantly superior AUPRC value of 0.903 compared to the UP model's AUPRC value of 0.385, with a P-value less than 0.05.

**Table 6:** Performance of the optimal UP (PD_ave,q1_) and LR model.

	AUROC	Opt. cutoff	Sensitivity	Specificity	AUPRC
UP	0.747 (0.573–0.889)	0.530	0.889	0.614	0.385 (0.142–0.647)
LR	0.890 (0.843–0.932)	0.718	0.719	0.877	0.903 (0.848–0.945)

## 4 Discussion

This study compared the performance of predictive models based on UP and LR using 91 spatiotemporal features derived from seven hemodynamic parameters. The results of UP showed a wide range of AUROC values (ranging from 0.224 to 0.747) for different spatiotemporal hemodynamic features. Notably, 22 pairs of features demonstrated significant differences in AUROC values with P-value <0.05, even though they were derived from the same hemodynamic parameter. Among the examined parameters, PD was found to have the highest performance, with PD_ave,q1_ being the strongest UP with AUROC/AUPRC values of 0.747/0.385, yielding sensitivity and specificity value of 0.889 and 0.614 at the optimal cut-off value, respectively. The LR model further improved the prediction performance, having AUROC/AUPRC values of 0.890/0.903. At the optimal cut-off value, the LR model achieved a specificity of 0.877, sensitivity of 0.719, outperforming the UP model.

Identifying the factors that contribute to intracranial aneurysm recanalization is crucial for physicians. However, contradictory findings have been reported due to uncertainties in CFD, including inaccurate geometry, missing boundary conditions, and unclear model parameters [[24], [25], [26], [27], [28], [29]]. The impact of spatiotemporal definition on the uncertainty has not been thoroughly investigated. Our study revealed that the spatiotemporal definition method employed for hemodynamic parameters had a substantial impact on the performance of predictive models. This finding led to the hypothesis that distinct spatiotemporal features might be associated with different pathologies of aneurysm recanalization. For instance, the identification of volvel_max,max_ as a significant risk factor suggested that an elevated velocity at a specific spatial and temporal point might contribute to an increased likelihood of aneurysm recanalization. Conversely, the identification of volvel_ave,max_ as a risk factor indicated that a high velocity throughout the aneurysm dome at a particular time point might be associated with the occurrence of recanalization. Similarly, the identification of volvel_max,a_ as a risk factor signified that a high velocity at a specific point over time might be associated with an increased risk of aneurysm recanalization. On the other hand, the identification of volvel_ave,a_ as a risk factor suggested that a high velocity throughout the aneurysm dome over time might significantly influence the likelihood of recanalization. This study observed the prevalence of spatial averaging over maximizing in both UP and LR analyses: (1) eight of top ten most effective UP; (2) five in seven hemodynamic features selected by VIF analysis; (3) all three hemodynamic features in the final LR model were spatially averaged. It suggested that a higher velocity/pressure difference distributed throughout the aneurysm dome/neck might pose a greater risk for aneurysm recanalization compared to a high velocity/pressure difference localized to a single location.

Furthermore, this superiority of spatial averaging might also be attributed to the inherent robustness of this method in processing hemodynamic parameters. Validation studies of *in vitro*, *in vivo* and multi-modalities, indicated that global data, such as flow patterns and spatially averaged hemodynamic parameters, showed higher robustness than point-wise data, especially under complex flow conditions [[30], [31], [32], [33], [34]]. Flow disturbances and irregularities result in irrelevant data, which indicates that averaged data are superior to point-wise data [30,33].

Compared to spatial characteristics, temporal characteristics preferred to be described with multiple features: (1) various PD temporal features occupied top ten best UPs; (2) temporal features except averaged and maximum remained after univariate logistic and VIF analysis; (2) the final LR analysis selected two types of temporal features derived from PD, namely, std and min. Recently, time series data was utilized to develop prediction models for cardiovascular diseases [35,36]. However, only physiological time series, such as blood pressure, heart rate and ECG were used. Our study indicates that the temporal characteristics of hemodynamic parameters derived from transient CFD simulations could offer clinically interpretable and significant information for identifying patients with a high likelihood of recanalization. To further investigate the potential of these time series features, we plan to extract more hemodynamic features in 3D space and time using deep learning and analyze additional features in the frequency and wavelet domains using a larger dataset [9,[35], [36], [37], [38]].

The process of deriving spatiotemporal features resulted in a rapid increase in the number of features, which in turn led to increased complexity of the classifiers and overfitting caused by irrelevant and meaningless data [39]. To address this issue, we focused on selecting only the most significant and non-multicollinear features to improve model performance. The resulting LR model achieved satisfactory results on the current dataset, with an AUROC value of 0.890. In future research, we plan to examine additional significant features for predicting aneurysm recanalization and evaluate the model on a larger and external dataset.

Currently, there are two primary methods for acquiring hemodynamics data from patients in clinical settings: CFD or non-invasive measurements using techniques such as 4D-Flow MRI or Transcranial Doppler Ultrasound [40,41]. The utilization of CFD has been limited in clinical practice due to its intricate processing requirements and time-consuming calculations. Among the experimental methodologies, only 4D-Flow MRI furnishes comprehensive volumetric data pertaining to time-resolved flow patterns [42]. In addition, 4D-Flow MRI enables relatively faster acquisition of patient-specific hemodynamics without the need for simplifying model parameters and boundary conditions, in contrast to the CFD method. Although 4D-Flow MRI exhibits lower resolution in small cerebral arteries, recent advancements in data assimilation techniques have demonstrated promising outcomes in enhancing the resolution of 4D-Flow MRI. As a result, it holds significant potential as an approach for obtaining spatiotemporal hemodynamic features in clinical practice in the foreseeable future [42,43].

The study confirmed again the superior performance of PD over other hemodynamic parameters, as nine out of the top ten UPs with the highest AUROC values and two of the three hemodynamic features selected by LR model were derived from PD. Additionally, the study enhanced the predictive performance of PD by exploring the impact of spatiotemporal characteristics, ultimately identifying PD_ave,q1_ as the most robust predictor with AUROC value of 0.747. In contrast, the previously published parameter PD_max,max_ demonstrated a lower AUROC value of 0.649 [1,2,8].

This study did not consider the coiling configuration. The coil surface was depicted as a planar and rigid structure; in contrast, clinical reality often involves a textured coil surface that facilitates blood permeation into the coil mass. Further investigation is needed to integrate the realistic coil surface obtained through advanced techniques such as silent magnetic resonance angiography into the analysis [44].

The technique SMOTE was applied to rebalance the data by resampling minority cases. While SMOTE has found application in numerous medical studies, it is important to acknowledge that this method generates synthetic samples, potentially introducing an influence on the outcomes [[45], [46], [47]].

## 5 Limitations

First, this study involved a cohort of 65 patients. Future steps will encompass an expanded analysis with a larger patient cohort for further evaluation. Second, given its retrospective nature, subsequent research should focus on a prospective study involving a comprehensive cohort covering all types of aneurysms to validate the predictive effectiveness of the developed models. Third, the present study did not account for fluid-solid interaction between vessel walls and blood. Finally, uniform boundary conditions were applied to all patients. Future investigations will explore the feasibility of integrating patient-specific boundary conditions derived from advanced modalities such as 4D-Flow MRI or Transcranial Doppler Ultrasonography to enhance accuracy.

## 6 Conclusion

The study demonstrated that the model performance in predicting aneurysm recanalization was significantly influenced by the spatiotemporal characteristics of hemodynamic parameters. PD_ave,q1_ was the strongest UP. The performance of LR model was enhanced by incorporating multiple features to describe temporal characteristics and utilizing spatial averaging. This enhanced predictive model holds clinical promise for non-invasively predicting recanalization following coil embolization in patients.

## 7 Ethics approval

The retrospective study protocol received approval from the Ethics Committee of Kanazawa University (Approval No. 1781). Given the retrospective nature of clinical data collection, the requirement for written informed consent was waived. Nevertheless, all patients retained the prerogative to withdraw their participation from the study at any given point in time.

## Grant support

This work was partially supported by JST SPRING (Grant Number JPMJSP2135 to L.J.) and The 10.13039/501100001691Japan Society for the Promotion of Science (10.13039/501100001691JSPS) KAKENHI (Grant Numbers C–16K10783 to K.M.)

## CRediT authorship contribution statement

**Jing Liao:** Conceptualization, Data curation, Formal analysis, Funding acquisition, Investigation, Methodology, Project administration, Resources, Software, Supervision, Validation, Visualization, Writing - original draft, Writing - review & editing. **Kouichi Misaki:** Data curation, Funding acquisition, Project administration, Resources, Supervision, Validation, Writing - review & editing, Software. **Tekehiro Uno:** Data curation, Resources. **Iku Nambu:** Data curation, Resources. **Tomoya Kamide:** Data curation, Resources. **Chen Zhuoqing:** Conceptualization, Investigation, Methodology. **Mitsutoshi Nakada:** Data curation, Funding acquisition, Project administration, Resources, Software, Supervision. **Jiro Sakamoto:** Project administration, Resources, Supervision.

## Declaration of competing interest

This work was partially supported by 10.13039/501100002241Japan Science and Technology Agency Support for Pioneering Research Initiated by the Next Generation (JST SPRING) (Grant Number JPMJSP2135 to L.J.) and The 10.13039/501100001691Japan Society for the Promotion of Science (10.13039/501100001691JSPS) KAKENHI (Grant Numbers C–16K10783 to K.M.)

The authors declare that they have no known competing financial interests or personal relationships that could have appeared to influence the work reported in this paper.
