# Supplementary material for: Fluid dynamic analysis in predicting the recanalization of intracranial aneurysms after coil embolization – A study of spatiotemporal characteristics
Source: Heliyon. 2023 Dec 20;10(1):e22801. doi: 10.1016/j.heliyon.2023.e22801 (PMC10788401; doi:10.1016/j.heliyon.2023.e22801)
Supplement: Multimedia component 1 [file mmc1.docx]

Supplementary Table 1 Parametric or nonparametric test for all morphological and hemodynamic features

|  | Stable (N = 57) | Recanalized (N = 9) | P-value |
| --- | --- | --- | --- |
| Age | 64.649±12.749 | 64.556±12.157 | 0.984 |
| Max size | 7.987±3.135 | 11.344±4.33 | 0.007 |
| Neck width | 4.805±1.463 | 6.311±2.353 | 0.112 |
| Aspect ratio | 1.275±0.482 | 1.411±0.489 | 0.440 |
| Bottleneck | 1.591±0.631 | 1.732±0.564 | 0.535 |
| Height | 5.967±2.63 | 8.444±3.855 | 0.019 |
| Size ratio | 1.969±0.873 | 2.457±1.025 | 0.139 |
| Diam Inflow | 2.555±1.134 | 2.534±1.187 | 0.959 |
| Area Inflow | 9.357±4.195 | 11.848±6.148 | 0.134 |
| Area Neck | 21.813±13.745 | 34.336±19.49 | 0.022 |
| Area Ratio | 0.497±0.23 | 0.385±0.16 | 0.173 |
| Pcom | 1.06±0.868 | 1.672±1.245 | 0.075 |
| Area Pcom | 1.475±1.587 | 3.415±3.682 | 0.178 |
| VER | 24.663±5.17 | 20.967±7.97 | 0.076 |
| volvelave,a | 0.441±0.159 | 0.299±0.161 | 0.017 |
| volvelave,std | 0.036±0.012 | 0.032±0.017 | 0.386 |
| volvelave,min | 0.327±0.134 | 0.195±0.107 | 0.007 |
| volvelave,q1 | 0.424±0.158 | 0.288±0.156 | 0.021 |
| volvelave,median | 0.443±0.16 | 0.303±0.162 | 0.020 |
| volvelave,q2 | 0.462±0.161 | 0.316±0.169 | 0.016 |
| volvelave,max | 0.513±0.17 | 0.357±0.193 | 0.016 |
| volvelmax,a | 1.444±0.26 | 1.776±1.296 | 0.490 |
| volvelmax,std | 0.051±0.021 | 0.095±0.1 | 0.249 |
| volvelmax,min | 1.264±0.249 | 1.474±1.088 | 0.601 |
| volvelmax,q1 | 1.427±0.262 | 1.736±1.221 | 0.495 |
| volvelmax,median | 1.45±0.262 | 1.786±1.281 | 0.481 |
| volvelmax,q2 | 1.47±0.262 | 1.829±1.356 | 0.477 |
| volvelmax,max | 1.535±0.268 | 1.995±1.663 | 0.457 |
| PDave,a | 0.361±0.623 | 0.903±0.765 | 0.024 |
| PDave,std | 0.073±0.031 | 0.102±0.044 | 0.017 |
| PDave,min | 0.091±0.638 | 0.503±0.714 | 0.086 |
| PDave,q1 | 0.33±0.638 | 0.882±0.774 | 0.025 |
| PDave,median | 0.368±0.628 | 0.922±0.774 | 0.023 |
| PDave,q2 | 0.404±0.615 | 0.948±0.77 | 0.022 |
| PDave,max | 0.488±0.591 | 1.056±0.779 | 0.015 |
| PDmax,a | 2.557±0.969 | 2.938±0.954 | 0.283 |
| PDmax,std | 0.181±0.08 | 0.234±0.056 | 0.065 |
| PDmax,min | 1.958±0.829 | 2.144±0.901 | 0.543 |
| PDmax,q1 | 2.495±0.955 | 2.869±0.926 | 0.284 |
| PDmax,median | 2.598±0.99 | 2.99±0.959 | 0.279 |
| PDmax,q2 | 2.67±1.007 | 3.079±1.004 | 0.269 |
| PDmax,max | 2.789±1.006 | 3.249±0.952 | 0.211 |
| Pave,a | 1.025±0.058 | 1.046±0.047 | 0.307 |
| Pave,std | 0.013±0.011 | 0.015±0.012 | 0.539 |
| Pave,min | 0.997±0.054 | 1.013±0.024 | 0.410 |
| Pave,q1 | 1.017±0.055 | 1.037±0.04 | 0.308 |
| Pave,median | 1.024±0.057 | 1.045±0.045 | 0.305 |
| Pave,q2 | 1.032±0.061 | 1.054±0.053 | 0.312 |
| Pave,max | 1.062±0.082 | 1.091±0.083 | 0.331 |
| Pmax,a | 1.192±0.206 | 1.135±0.059 | 0.424 |
| Pmax,std | 0.048±0.045 | 0.035±0.017 | 0.369 |
| Pmax,min | 1.084±0.11 | 1.056±0.029 | 0.445 |
| Pmax,q1 | 1.162±0.177 | 1.115±0.049 | 0.447 |
| Pmax,median | 1.186±0.201 | 1.132±0.058 | 0.428 |
| Pmax,q2 | 1.218±0.233 | 1.153±0.068 | 0.415 |
| Pmax,max | 1.334±0.346 | 1.236±0.109 | 0.409 |
| PPave,a | 0.044±0.046 | 0.055±0.037 | 0.528 |
| PPave,std | 0.012±0.012 | 0.015±0.012 | 0.519 |
| PPave,min | 0.018±0.024 | 0.021±0.015 | 0.771 |
| PPave,q1 | 0.036±0.039 | 0.045±0.03 | 0.512 |
| PPave,median | 0.043±0.044 | 0.053±0.036 | 0.521 |
| PPave,q2 | 0.051±0.052 | 0.063±0.043 | 0.540 |
| PPave,max | 0.081±0.077 | 0.099±0.074 | 0.524 |
| PPmax,a | 0.192±0.206 | 0.135±0.059 | 0.424 |
| PPmax,std | 0.048±0.045 | 0.035±0.017 | 0.369 |
| PPmax,min | 0.084±0.11 | 0.056±0.029 | 0.445 |
| PPmax,q1 | 0.162±0.177 | 0.115±0.049 | 0.447 |
| PPmax,median | 0.186±0.201 | 0.132±0.058 | 0.428 |
| PPmax,q2 | 0.218±0.233 | 0.153±0.068 | 0.415 |
| PPmax,max | 0.334±0.346 | 0.236±0.109 | 0.409 |
| Pdynave,a | 0.083±0.081 | 0.036±0.021 | 0.098 |
| Pdynave,std | 0.034±0.025 | 0.017±0.009 | 0.054 |
| Pdynave,min | 0.042±0.051 | 0.016±0.011 | 0.138 |
| Pdynave,q1 | 0.054±0.061 | 0.023±0.015 | 0.132 |
| Pdynave,median | 0.079±0.081 | 0.034±0.021 | 0.106 |
| Pdynave,q2 | 0.104±0.098 | 0.046±0.026 | 0.088 |
| Pdynave,max | 0.172±0.137 | 0.084±0.042 | 0.066 |
| Pdynmax,a | 0.877±0.534 | 0.574±0.329 | 0.108 |
| Pdynmax,std | 0.197±0.089 | 0.148±0.093 | 0.134 |
| Pdynmax,min | 0.568±0.433 | 0.341±0.223 | 0.134 |
| Pdynmax,q1 | 0.711±0.491 | 0.454±0.261 | 0.135 |
| Pdynmax,median | 0.876±0.541 | 0.571±0.337 | 0.111 |
| Pdynmax,q2 | 1.022±0.59 | 0.676±0.394 | 0.099 |
| Pdynmax,max | 1.34±0.651 | 0.928±0.502 | 0.079 |
| WSSave,a | 9.768±6.359 | 6.176±2.794 | 0.105 |
| WSSave,std | 3.584±2.069 | 2.366±1.107 | 0.094 |
| WSSave,min | 5.573±3.912 | 3.43±1.539 | 0.115 |
| WSSave,q1 | 6.791±4.653 | 4.235±1.901 | 0.114 |
| WSSave,median | 9.412±6.199 | 5.918±2.654 | 0.106 |
| WSSave,q2 | 11.833±7.586 | 7.451±3.379 | 0.098 |
| WSSave,max | 19.562±11.857 | 12.725±5.915 | 0.100 |
| WSSmax,a | 31.05±18.343 | 21.186±10.297 | 0.126 |
| WSSmax,std | 11.136±5.275 | 8.074±3.738 | 0.104 |
| WSSmax,min | 17.81±11.88 | 11.572±5.79 | 0.133 |
| WSSmax,q1 | 21.771±13.984 | 14.453±7.145 | 0.135 |
| WSSmax,median | 30.008±18.133 | 20.531±10.092 | 0.137 |
| WSSmax,q2 | 37.459±21.479 | 25.688±12.417 | 0.120 |
| WSSmax,max | 61.192±32.029 | 42.649±19.767 | 0.102 |
| FR,a | 0.00084±0.00077 | 0.00198±0.0038 | 0.051 |
| FR,std | 0.00023±0.00021 | 0.00033±0.00038 | 0.235 |
| FR,min | 0.00038±0.00058 | 0.00122±0.003 | 0.066 |
| FR,q1 | 0.0007±0.00071 | 0.00175±0.00349 | 0.050 |
| FR,median | 0.00081±0.00076 | 0.00195±0.0038 | 0.051 |
| FR,q2 | 0.00096±0.00084 | 0.00218±0.00407 | 0.051 |
| FR,max | 0.00166±0.00163 | 0.00287±0.00467 | 0.153 |

Supplementary Table 2 UP analysis

|  | AUCs | cfl | cfu | Opt. cutoff | sensitivity | specificity |
| --- | --- | --- | --- | --- | --- | --- |
| PDave,q1 | 0.747 | 0.573 | 0.889 | 0.530 | 0.889 | 0.614 |
| PDave,a | 0.743 | 0.571 | 0.884 | 0.541 | 0.889 | 0.614 |
| PDave,median | 0.743 | 0.569 | 0.885 | 0.556 | 0.889 | 0.614 |
| PDave,max | 0.742 | 0.570 | 0.891 | 0.640 | 0.889 | 0.632 |
| PDave,q2 | 0.742 | 0.569 | 0.886 | 0.574 | 0.889 | 0.614 |
| PDmax,std | 0.725 | 0.607 | 0.833 | 0.165 | 1.000 | 0.491 |
| PDave,min | 0.704 | 0.529 | 0.847 | 0.587 | 0.667 | 0.789 |
| PDave,std | 0.673 | 0.489 | 0.864 | 0.105 | 0.556 | 0.825 |
| PDmax,max | 0.649 | 0.464 | 0.810 | 3.270 | 0.667 | 0.702 |
| Pave,min | 0.641 | 0.476 | 0.789 | 1.004 | 0.889 | 0.474 |
| PPave,q1 | 0.641 | 0.504 | 0.777 | 0.016 | 1.000 | 0.386 |
| Pave,q1 | 0.639 | 0.477 | 0.793 | 1.016 | 0.889 | 0.509 |
| PDmax,q2 | 0.635 | 0.442 | 0.806 | 3.200 | 0.667 | 0.719 |
| PDmax,q1 | 0.635 | 0.438 | 0.811 | 3.040 | 0.667 | 0.719 |
| PDmax,median | 0.635 | 0.441 | 0.811 | 3.140 | 0.667 | 0.702 |
| PDmax,a | 0.632 | 0.436 | 0.806 | 3.075 | 0.667 | 0.719 |
| PPave,median | 0.628 | 0.483 | 0.772 | 0.019 | 1.000 | 0.351 |
| Pave,median | 0.624 | 0.458 | 0.780 | 1.019 | 0.889 | 0.474 |
| volvelmax,std | 0.624 | 0.466 | 0.789 | 0.044 | 0.889 | 0.404 |
| PPave,a | 0.622 | 0.476 | 0.769 | 0.019 | 1.000 | 0.351 |
| Pave,a | 0.618 | 0.455 | 0.775 | 1.019 | 0.889 | 0.474 |
| PPave,min | 0.618 | 0.463 | 0.756 | 0.004 | 1.000 | 0.298 |
| PPave,q2 | 0.616 | 0.459 | 0.767 | 0.022 | 1.000 | 0.333 |
| Pave,q2 | 0.610 | 0.447 | 0.772 | 1.022 | 0.889 | 0.456 |
| Pave,max | 0.591 | 0.419 | 0.765 | 1.030 | 0.889 | 0.351 |
| FR,std | 0.583 | 0.409 | 0.753 | 0.000 | 0.667 | 0.579 |
| FR,q1 | 0.579 | 0.428 | 0.729 | 0.000 | 0.778 | 0.474 |
| PPave,max | 0.573 | 0.411 | 0.750 | 0.030 | 1.000 | 0.246 |
| FR,min | 0.569 | 0.414 | 0.729 | 0.000 | 1.000 | 0.263 |
| PDmax,min | 0.563 | 0.361 | 0.744 | 3.120 | 0.222 | 0.947 |
| FR,a | 0.558 | 0.390 | 0.727 | 0.000 | 0.778 | 0.456 |
| FR,q2 | 0.554 | 0.380 | 0.721 | 0.001 | 0.778 | 0.526 |
| FR,max | 0.550 | 0.391 | 0.715 | 0.001 | 0.778 | 0.456 |
| FR,median | 0.548 | 0.379 | 0.710 | 0.000 | 0.778 | 0.439 |
| PPave,std | 0.536 | 0.357 | 0.723 | 0.016 | 0.444 | 0.754 |
| Pave,std | 0.532 | 0.347 | 0.723 | 0.016 | 0.444 | 0.754 |
| Pmax,q1 | 0.458 | 0.325 | 0.606 | 1.074 | 0.889 | 0.263 |
| PPmax,q1 | 0.458 | 0.325 | 0.606 | 0.074 | 0.889 | 0.263 |
| volvelave,std | 0.456 | 0.245 | 0.673 | 0.042 | 0.444 | 0.754 |
| volvelmax,max | 0.442 | 0.255 | 0.641 | 6.659 | 0.111 | 1.000 |
| volvelmax,median | 0.439 | 0.247 | 0.646 | 1.780 | 0.222 | 0.912 |
| PPmax,a | 0.439 | 0.305 | 0.590 | 0.086 | 0.889 | 0.246 |
| Pmax,a | 0.439 | 0.305 | 0.590 | 1.086 | 0.889 | 0.246 |
| volvelmax,q1 | 0.435 | 0.247 | 0.639 | 1.753 | 0.222 | 0.895 |
| volvelmax,q2 | 0.433 | 0.240 | 0.637 | 1.786 | 0.222 | 0.912 |
| Pmax,median | 0.433 | 0.295 | 0.588 | 1.083 | 0.889 | 0.228 |
| PPmax,median | 0.433 | 0.295 | 0.588 | 0.083 | 0.889 | 0.228 |
| PPmax,q2 | 0.431 | 0.293 | 0.588 | 0.096 | 0.889 | 0.246 |
| Pmax,q2 | 0.431 | 0.293 | 0.588 | 1.096 | 0.889 | 0.246 |
| volvelmax,a | 0.429 | 0.236 | 0.637 | 1.757 | 0.222 | 0.895 |
| PPmax,min | 0.427 | 0.271 | 0.593 | 0.036 | 0.889 | 0.228 |
| Pmax,min | 0.427 | 0.271 | 0.593 | 1.036 | 0.889 | 0.228 |
| PPmax,std | 0.409 | 0.251 | 0.575 | 0.013 | 1.000 | 0.088 |
| Pmax,std | 0.409 | 0.251 | 0.575 | 0.013 | 1.000 | 0.088 |
| Pmax,max | 0.407 | 0.258 | 0.567 | 1.123 | 1.000 | 0.123 |
| PPmax,max | 0.407 | 0.258 | 0.567 | 0.123 | 1.000 | 0.123 |
| volvelmax,min | 0.398 | 0.203 | 0.613 | 4.491 | 0.111 | 1.000 |
| Pdynmax,std | 0.341 | 0.142 | 0.541 | 0.336 | 0.111 | 0.947 |
| Pdynmax,q1 | 0.335 | 0.172 | 0.500 | 0.145 | 1.000 | 0.018 |
| Pdynmax,median | 0.312 | 0.146 | 0.491 | 3.852 | 0.000 | 1.000 |
| WSSmax,median | 0.306 | 0.126 | 0.495 | 9.025 | 1.000 | 0.018 |
| Pdynmax,a | 0.306 | 0.137 | 0.489 | 3.838 | 0.000 | 1.000 |
| Pdynmax,min | 0.306 | 0.144 | 0.475 | 3.157 | 0.000 | 1.000 |
| volvelave,max | 0.300 | 0.146 | 0.471 | 1.991 | 0.000 | 1.000 |
| Pdynmax,max | 0.300 | 0.128 | 0.479 | 4.484 | 0.000 | 1.000 |
| WSSmax,a | 0.294 | 0.111 | 0.484 | 9.348 | 1.000 | 0.018 |
| WSSmax,q1 | 0.294 | 0.118 | 0.474 | 7.007 | 1.000 | 0.035 |
| WSSmax,q2 | 0.292 | 0.116 | 0.481 | 115.422 | 0.000 | 1.000 |
| WSSave,min | 0.290 | 0.123 | 0.462 | 1.876 | 1.000 | 0.018 |
| WSSmax,std | 0.290 | 0.109 | 0.478 | 29.696 | 0.000 | 1.000 |
| WSSave,q1 | 0.288 | 0.121 | 0.462 | 2.205 | 1.000 | 0.018 |
| volvelave,median | 0.288 | 0.134 | 0.454 | 1.902 | 0.000 | 1.000 |
| volvelave,q1 | 0.288 | 0.138 | 0.450 | 1.883 | 0.000 | 1.000 |
| WSSmax,min | 0.288 | 0.111 | 0.466 | 5.934 | 1.000 | 0.035 |
| volvelave,q2 | 0.287 | 0.132 | 0.452 | 1.921 | 0.000 | 1.000 |
| volvelave,a | 0.285 | 0.131 | 0.452 | 1.901 | 0.000 | 1.000 |
| WSSave,median | 0.285 | 0.119 | 0.455 | 2.977 | 1.000 | 0.018 |
| Pdynmax,q2 | 0.285 | 0.119 | 0.464 | 4.117 | 0.000 | 1.000 |
| WSSave,max | 0.283 | 0.111 | 0.460 | 5.833 | 1.000 | 0.018 |
| Pdynave,q1 | 0.283 | 0.140 | 0.431 | 0.008 | 1.000 | 0.035 |
| WSSave,q2 | 0.281 | 0.113 | 0.455 | 3.509 | 1.000 | 0.018 |
| WSSave,a | 0.281 | 0.116 | 0.453 | 3.039 | 1.000 | 0.018 |
| WSSmax,max | 0.281 | 0.098 | 0.472 | 172.263 | 0.000 | 1.000 |
| WSSave,std | 0.279 | 0.106 | 0.452 | 11.289 | 0.000 | 1.000 |
| Pdynave,min | 0.277 | 0.140 | 0.419 | 0.006 | 1.000 | 0.053 |
| Pdynave,median | 0.255 | 0.128 | 0.389 | 0.010 | 1.000 | 0.018 |
| Pdynave,a | 0.250 | 0.125 | 0.385 | 0.010 | 1.000 | 0.018 |
| Pdynave,q2 | 0.244 | 0.119 | 0.362 | 0.011 | 1.000 | 0.018 |
| Pdynave,max | 0.236 | 0.116 | 0.352 | 0.024 | 1.000 | 0.018 |
| Pdynave,std | 0.234 | 0.111 | 0.357 | 0.004 | 1.000 | 0.018 |
| volvelave,min | 0.224 | 0.099 | 0.370 | 1.741 | 0.000 | 1.000 |

Supplementary Table 3 Univariate logistic regression

| Features | Odds Ratio | 5% CI | 95% CI | P value |
| --- | --- | --- | --- | --- |
| Pdynave,std | 3.797E-49 | 4.903E-51 | 2.941E-47 | 3.941E-06 |
| Max size | 1.310E+00 | 1.282E+00 | 1.339E+00 | 8.568E-06 |
| Pdynave,max | 9.706E-10 | 4.389E-10 | 2.146E-09 | 1.178E-05 |
| Height | 1.343E+00 | 1.305E+00 | 1.382E+00 | 4.016E-05 |
| WSSmax,max | 9.491E-01 | 9.456E-01 | 9.526E-01 | 9.222E-05 |
| WSSave,max | 8.405E-01 | 8.328E-01 | 8.482E-01 | 1.012E-04 |
| WSSave,q1 | 6.081E-01 | 5.940E-01 | 6.225E-01 | 1.420E-04 |
| WSSave,q2 | 7.569E-01 | 7.465E-01 | 7.675E-01 | 1.506E-04 |
| WSSave,min | 5.512E-01 | 5.360E-01 | 5.669E-01 | 1.591E-04 |
| WSSmax,min | 8.562E-01 | 8.474E-01 | 8.651E-01 | 2.024E-04 |
| WSSave,std | 4.307E-01 | 4.086E-01 | 4.540E-01 | 2.228E-04 |
| WSSmax,std | 7.791E-01 | 7.621E-01 | 7.964E-01 | 2.275E-04 |
| WSSmax,q1 | 8.820E-01 | 8.743E-01 | 8.897E-01 | 2.345E-04 |
| WSSave,a | 7.304E-01 | 7.183E-01 | 7.426E-01 | 2.726E-04 |
| WSSave,median | 7.239E-01 | 7.116E-01 | 7.364E-01 | 2.955E-04 |
| WSSmax,a | 9.190E-01 | 9.131E-01 | 9.250E-01 | 3.364E-04 |
| Pdynave,q1 | 1.134E-19 | 1.129E-20 | 1.138E-18 | 3.733E-04 |
| WSSmax,median | 9.203E-01 | 9.142E-01 | 9.263E-01 | 4.515E-04 |
| Pdynave,a | 4.991E-13 | 1.022E-13 | 2.437E-12 | 4.840E-04 |
| Pdynave,min | 1.038E-24 | 6.908E-26 | 1.558E-23 | 5.224E-04 |
| volvelave,max | 1.533E-02 | 8.740E-03 | 2.690E-02 | 5.445E-04 |
| Pdynmax,a | 1.316E-01 | 1.027E-01 | 1.687E-01 | 7.536E-04 |
| Pdynave,median | 1.469E-12 | 2.997E-13 | 7.204E-12 | 7.560E-04 |
| volvelave,a | 1.240E-02 | 6.541E-03 | 2.349E-02 | 1.033E-03 |
| Pdynmax,min | 6.838E-02 | 5.005E-02 | 9.342E-02 | 1.220E-03 |
| volvelave,q1 | 1.313E-02 | 6.832E-03 | 2.522E-02 | 1.406E-03 |
| Pdynmax,median | 1.729E-01 | 1.358E-01 | 2.202E-01 | 1.800E-03 |
| WSSmax,q2 | 9.481E-01 | 9.429E-01 | 9.533E-01 | 1.803E-03 |
| PDmax,std | 9.781E+03 | 2.428E+03 | 3.940E+04 | 1.907E-03 |
| Area Ratio | 2.601E-02 | 1.507E-02 | 4.491E-02 | 2.184E-03 |
| volvelmax,std | 6.878E+04 | 1.906E+04 | 2.482E+05 | 2.568E-03 |
| volvelmax,q1 | 2.760E+00 | 2.443E+00 | 3.119E+00 | 3.959E-03 |
| volvelmax,median | 2.659E+00 | 2.369E+00 | 2.983E+00 | 4.205E-03 |
| volvelmax,a | 2.600E+00 | 2.320E+00 | 2.914E+00 | 4.368E-03 |
| volvelmax,q2 | 2.600E+00 | 2.332E+00 | 2.899E+00 | 4.459E-03 |
| volvelmax,min | 2.389E+00 | 2.073E+00 | 2.753E+00 | 6.486E-03 |
| Pdynave,q2 | 5.090E-06 | 1.503E-06 | 1.723E-05 | 1.168E-02 |
| Pdynmax,q2 | 3.362E-01 | 2.706E-01 | 4.178E-01 | 1.307E-02 |
| PDave,min | 2.118E+00 | 1.799E+00 | 2.494E+00 | 1.417E-02 |
| VER | 9.447E-01 | 9.305E-01 | 9.591E-01 | 3.971E-02 |
| PDave,std | 5.959E+04 | 3.733E+03 | 9.511E+05 | 4.019E-02 |
| Pave,median | 5.896E+03 | 5.103E+02 | 6.812E+04 | 4.591E-02 |
| PDmax,median | 1.500E+00 | 1.328E+00 | 1.694E+00 | 5.251E-02 |
| PDmax,q1 | 1.512E+00 | 1.332E+00 | 1.715E+00 | 5.606E-02 |
| Aspect ratio | 2.241E+00 | 1.777E+00 | 2.827E+00 | 5.942E-02 |
| Pave,q1 | 6.637E+03 | 5.053E+02 | 8.716E+04 | 6.456E-02 |
| Pave,a | 1.992E+03 | 1.847E+02 | 2.149E+04 | 6.627E-02 |
| FR,max | 2.261E+54 | 3.790E+37 | 1.349E+71 | 6.663E-02 |
| Pave,q2 | 8.532E+02 | 9.247E+01 | 7.873E+03 | 7.073E-02 |
| volvelave,std | 3.820E-12 | 1.935E-16 | 7.543E-08 | 7.801E-02 |
| FR,q1 | 1.568E+70 | 3.003E+46 | 8.189E+93 | 8.774E-02 |
| Pave,min | 1.897E+05 | 1.176E+04 | 3.061E+06 | 9.276E-02 |
| Area Neck | 1.019E+00 | 1.013E+00 | 1.026E+00 | 9.327E-02 |
| PDave,max | 1.579E+00 | 1.357E+00 | 1.837E+00 | 1.006E-01 |
| PDave,median | 1.553E+00 | 1.348E+00 | 1.789E+00 | 1.049E-01 |
| PDave,q1 | 1.547E+00 | 1.344E+00 | 1.781E+00 | 1.063E-01 |
| PDave,q2 | 1.549E+00 | 1.341E+00 | 1.788E+00 | 1.120E-01 |
| PDave,a | 1.543E+00 | 1.337E+00 | 1.781E+00 | 1.143E-01 |
| Pave,max | 3.246E+01 | 6.640E+00 | 1.586E+02 | 1.590E-01 |
| PPave,q1 | 3.615E+03 | 9.433E+01 | 1.386E+05 | 1.670E-01 |
| Location,1 | 4.178E-01 | 2.769E-01 | 6.305E-01 | 1.994E-01 |
| Pdynmax,std | 7.093E-02 | 2.001E-02 | 2.515E-01 | 2.095E-01 |
| Pdynmax,max | 6.689E-01 | 5.540E-01 | 8.077E-01 | 2.125E-01 |
| volvelave,q2 | 2.642E-01 | 1.419E-01 | 4.920E-01 | 2.323E-01 |
| PPave,median | 3.936E+02 | 1.666E+01 | 9.297E+03 | 2.350E-01 |
| PPave,a | 2.701E+02 | 1.253E+01 | 5.822E+03 | 2.498E-01 |
| volvelave,median | 2.756E-01 | 1.457E-01 | 5.215E-01 | 2.587E-01 |
| PDmax,a | 1.260E+00 | 1.110E+00 | 1.429E+00 | 2.590E-01 |
| Pave,std | 3.474E+07 | 2.923E+02 | 4.128E+12 | 3.187E-01 |
| volvelave,min | 2.723E-01 | 1.268E-01 | 5.847E-01 | 3.534E-01 |
| PPave,max | 1.118E+01 | 1.938E+00 | 6.448E+01 | 3.641E-01 |
| Rupture | 6.318E-01 | 4.481E-01 | 8.906E-01 | 3.789E-01 |
| PPave,std | 3.456E+06 | 3.076E+01 | 3.882E+11 | 3.832E-01 |
| volvelmax,max | 1.131E+00 | 1.037E+00 | 1.234E+00 | 3.947E-01 |
| PDmax,min | 1.216E+00 | 1.036E+00 | 1.428E+00 | 3.975E-01 |
| Pmax,max | 4.943E-01 | 3.011E-01 | 8.115E-01 | 4.138E-01 |
| PPmax,max | 4.943E-01 | 3.011E-01 | 8.115E-01 | 4.138E-01 |
| Location,3 | 2.853E-01 | 1.060E-01 | 7.682E-01 | 4.614E-01 |
| Location,2 | 2.853E-01 | 1.060E-01 | 7.682E-01 | 4.614E-01 |
| PPave,q2 | 1.788E+01 | 1.220E+00 | 2.620E+02 | 4.872E-01 |
| Bottleneck | 1.251E+00 | 1.037E+00 | 1.511E+00 | 4.962E-01 |
| FR,q2 | 1.039E+20 | 1.930E+00 | 5.600E+39 | 5.067E-01 |
| Pmax,min | 1.728E-01 | 3.892E-02 | 7.673E-01 | 5.092E-01 |
| PPmax,min | 1.728E-01 | 3.892E-02 | 7.673E-01 | 5.092E-01 |
| Pdynmax,q1 | 7.484E-01 | 5.650E-01 | 9.914E-01 | 5.381E-01 |
| Area Inflow | 1.024E+00 | 9.976E-01 | 1.052E+00 | 5.447E-01 |
| PPave,min | 3.270E+02 | 7.930E-01 | 1.348E+05 | 5.476E-01 |
| FR,a | 1.395E+19 | 5.758E-03 | 3.381E+40 | 5.524E-01 |
| FR,median | 1.047E+19 | 2.901E-03 | 3.778E+40 | 5.544E-01 |
| Sex,1 | 7.682E-01 | 5.841E-01 | 1.010E+00 | 5.564E-01 |
| Sex,0 | 6.486E-01 | 3.935E-01 | 1.069E+00 | 5.623E-01 |
| Size ratio | 1.114E+00 | 9.937E-01 | 1.248E+00 | 5.633E-01 |
| PPmax,q2 | 5.631E-01 | 2.726E-01 | 1.163E+00 | 6.187E-01 |
| Pmax,q2 | 5.631E-01 | 2.726E-01 | 1.163E+00 | 6.187E-01 |
| PPmax,a | 5.949E-01 | 2.616E-01 | 1.353E+00 | 6.846E-01 |
| Pmax,a | 5.949E-01 | 2.616E-01 | 1.353E+00 | 6.846E-01 |
| Age | 1.007E+00 | 9.945E-01 | 1.020E+00 | 6.891E-01 |
| FR,min | 7.626E+15 | 2.670E-12 | 2.179E+43 | 6.955E-01 |
| PPmax,median | 6.055E-01 | 2.607E-01 | 1.407E+00 | 7.009E-01 |
| Pmax,median | 6.055E-01 | 2.607E-01 | 1.407E+00 | 7.009E-01 |
| Neck width | 1.033E+00 | 9.778E-01 | 1.091E+00 | 7.298E-01 |
| PDmax,max | 1.068E+00 | 9.478E-01 | 1.203E+00 | 7.362E-01 |
| PDmax,q2 | 1.061E+00 | 9.390E-01 | 1.199E+00 | 7.588E-01 |
| PPmax,q1 | 6.444E-01 | 2.463E-01 | 1.686E+00 | 7.649E-01 |
| Pmax,q1 | 6.444E-01 | 2.463E-01 | 1.686E+00 | 7.649E-01 |
| Area Pcom | 1.020E+00 | 9.631E-01 | 1.080E+00 | 8.188E-01 |
| Pmax,std | 3.161E-01 | 7.941E-03 | 1.259E+01 | 8.357E-01 |
| PPmax,std | 3.161E-01 | 7.941E-03 | 1.259E+01 | 8.357E-01 |
| Pcom | 1.037E+00 | 8.984E-01 | 1.198E+00 | 8.558E-01 |
| FR,std | 3.475E+14 | 7.515E-176 | 1.607E+204 | 9.591E-01 |
| Diam Inflow | 1.000E+00 | 8.940E-01 | 1.119E+00 | 9.991E-01 |
| Location,0 | 1.000E+00 | 7.336E-01 | 1.363E+00 | 9.998E-01 |

| Supplementary Table 4. Results of univariate logistic and VIF analysis | | | | | |
| --- | --- | --- | --- | --- | --- |
|  | Odds Ratio | 5% CI | 95% CI | P-value | VIF |
| Area Ratio | 0.026 | 0.015 | 0.045 | 0.002 | 6.429 |
| volvelave,q1 | 0.013 | 0.007 | 0.025 | 0.001 | 6.855 |
| volvelmax,std | 6.878E+04 | 1.906E+04 | 2.482E+05 | 0.003 | 2.247 |
| PDave,std | 5.959E+04 | 3.733E+03 | 9.511E+05 | 0.040 | 5.078 |
| PDave,min | 2.118 | 1.799 | 2.494 | 0.014 | 1.374 |
| PDYNave,min | 1.038E-24 | 6.908E-26 | 1.558E-23 | 0.001 | 6.213 |
| PDYNmax,q2 | 0.336 | 0.271 | 0.418 | 0.013 | 6.448 |
| WSSave,min | 0.551 | 0.536 | 0.567 | 0.000 | 9.217 |
